# Nephrostomy-Assisted Multimodal Removal of an Embedded Ureteral Metallic Stent: A Case Report

**DOI:** 10.7759/cureus.98203

**Published:** 2025-11-30

**Authors:** Tomohiro Matsuo, Kyohei Araki, Hiroyuki Honda, Kensuke Mitsunari, Ryoichi Imamura

**Affiliations:** 1 Department of Urology, Nagasaki University Hospital, Nagasaki, JPN; 2 Department of Urology, Graduate School of Biomedical Sciences, Nagasaki University, Nagasaki, JPN

**Keywords:** antegrade–retrograde approach, embedded stent, holmium:yag laser, metallic stent, percutaneous nephrostomy

## Abstract

In malignant ureteral obstruction (MUO), metallic ureteral stents are widely used to maintain urinary drainage; however, prolonged indwelling can lead to tissue ingrowth and encrustation, complicating safe removal. Here, we report a case in which standard retrograde removal was considered unsafe due to stent embedding. A female patient aged 45 years presented with a ureteral metallic stent that could not be safely removed via the transurethral route. To enable controlled removal, a percutaneous nephrostomy was created to establish antegrade access. A stepwise, multimodal strategy was employed: initial traction to evaluate mobility, holmium:YAG laser incision to release ingrowth, and careful dissection with endoscopic scissors to preserve ureteral integrity. The stent was removed en bloc without structural injury. This case demonstrates the effectiveness and safety of a nephrostomy-assisted multimodal method for embedded ureteral metallic stents. When conventional retrograde removal is unsuccessful or unsafe, combining antegrade access with traction, holmium laser release, and endoscopic scissor dissection can enable definitive explantation while limiting urinary tract injury.

## Introduction

Malignant ureteral obstruction (MUO) is a common sequela of advanced malignancies and can lead to hydronephrosis, flank pain, infection, and progressive loss of renal function. Metallic ureteral stents are increasingly used to maintain urinary drainage and preserve renal function, especially when frequent exchanges are expected [1-3]. Recent evidence indicates that metallic ureteral stents provide favorable long-term patency and lower rates of migration or obstruction than polymeric double-J (DJ) ureteral stents. In a prospective Japanese multicenter cohort using the Resonance® metallic stent, the cumulative stent obstruction incidences at three, six, nine, and 12 months were 8.9%, 16.3%, 16.3%, and 18.8%, respectively, with preservation of renal function in most patients [2]. Other series of metallic designs have reported technical or clinical success rates of approximately 65-88%, with migration rates as low as 1% and obstruction rates of 6-17%, compared with higher failure rates for polymeric stents [1,3].

Nevertheless, uncommon complications such as heavy encrustation and tissue ingrowth or embedding can make endoscopic removal hazardous or unsuccessful; encrustation refers to bulky calcific deposits that accumulate on the stent surface, whereas tissue ingrowth or embedding describes incorporation of the stent into the urothelial wall by chronic inflammation and fibrosis [4]. In these difficult situations, a multidisciplinary, multimodal approach that integrates antegrade and retrograde access with holmium (Ho):YAG laser incision and mechanical dissection has been described to facilitate definitive removal while limiting ureteral injury [5-7].

Here, we report a case of nephrostomy-assisted multimodal removal of an embedded ureteral metallic stent in a patient with MUO, where stepwise traction, Ho:YAG laser release, and endoscopic scissor dissection enabled safe explantation after conventional retrograde removal was deemed unsafe, illustrating a structured, mechanism-oriented workflow for managing complex embedded metallic stents.

## Case presentation

A female patient aged 45 years with uterine cervical cancer underwent radical hysterectomy with pelvic lymphadenectomy in February 2022. Her initial postoperative course was uneventful; however, two months later, she developed bilateral hydronephrosis due to retroperitoneal nodal metastases. Bilateral polyurethane DJ ureteral stents were placed at another hospital in September 2022 and exchanged every three months. Because repeated visits were burdensome and the obstruction was malignant in origin, both stents were converted to metallic ureteral stents (Resonance®, Boston Scientific, Arden Hills, MN) in January 2023.

In February 2024, a routine exchange was attempted. The right stent was replaced without difficulty; however, the left stent could not be safely removed via the transurethral route. Suspecting proximal encrustation, two sessions of extracorporeal shock wave lithotripsy (ESWL) were performed. Despite this, multiple retrograde attempts were unsuccessful. Retrograde ureteroscopy allowed only limited visualization and revealed no intramural migration. The patient was referred to our institution in June 2024 for further management.

**Figure 1:**
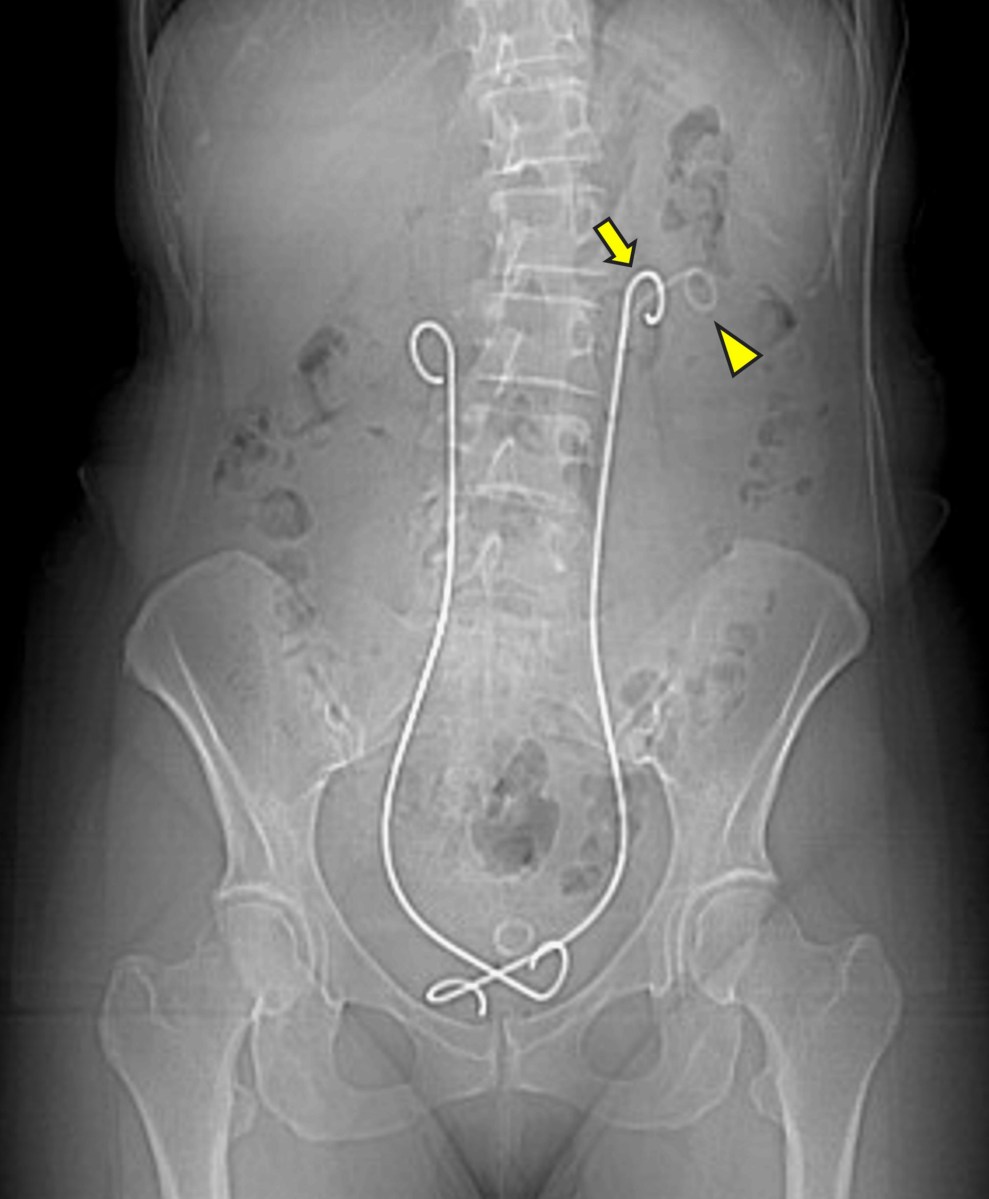
A plain abdominal radiograph at presentation to our institution. Metallic ureteral stent (arrow); polyurethane double-J (DJ) ureteral stents (arrowhead).

A plain radiograph obtained at the referring hospital showed bilateral metallic stents and an additional left-sided polyurethane DJ ureteral stent (Figure 1).

**Figure 2:**
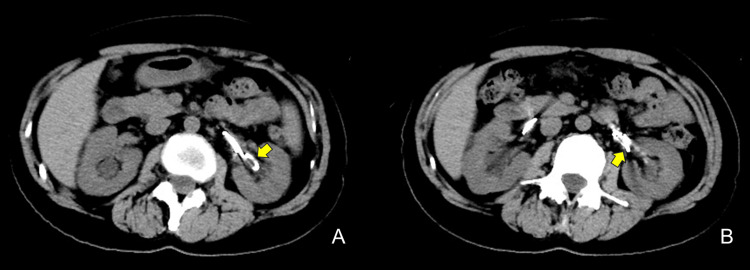
Non-contrast computed tomography (CT), axial images. A: Polyurethane double-J (DJ) ureteral stent (arrow). B: Metallic ureteral stent (arrow).

Computed tomography (CT) demonstrated no hydronephrosis of the left kidney and no calculi in the urinary tract or stents (Figures 2A, 2B).

**Figure 3:**
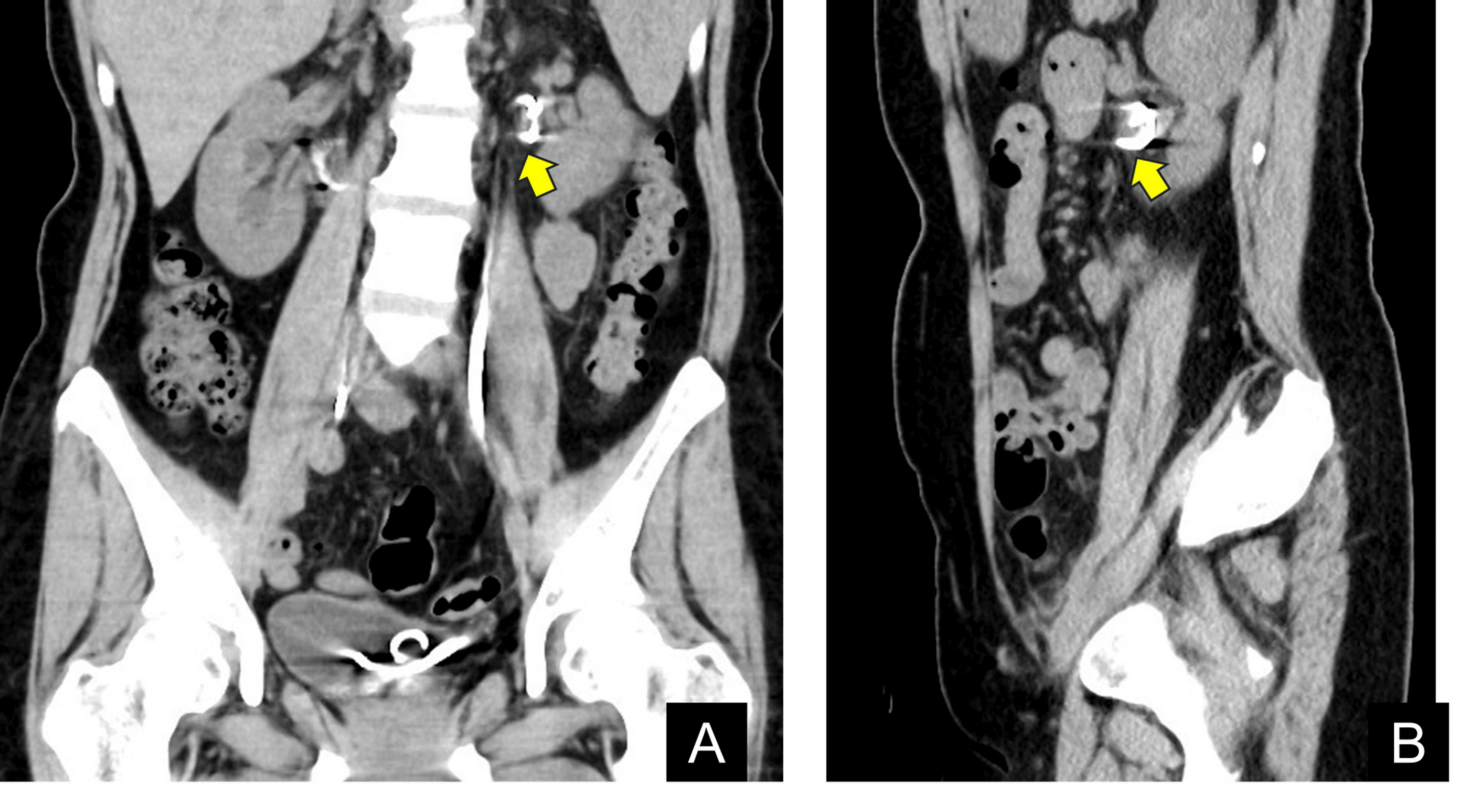
Non-contrast computed tomography (CT) findings. Because of a contrast media allergy, only a non-contrast CT was obtained; the images suggest possible embedding of the proximal loop of the metallic stent within the renal pelvic mucosa (arrows). A: Coronal image. B: Sagittal image.

The proximal loop of the left metallic stent appeared embedded within the renal pelvic mucosa (Figures 3A, 3B).

In July 2024, the patient underwent stent removal under general anesthesia. With the patient in the Galdakao-modified supine Valdivia (GMSV) position, a percutaneous nephrostomy was established. The tract was dilated using a 24-Fr high-pressure balloon dilator (NephroMax™ High-Pressure Nephrostomy Balloon Catheters Kit, Boston Scientific), and a 21-Fr nephroscope was introduced. The polyurethane DJ ureteral stent was removed first using 5-Fr nephroscopic forceps to improve visualization. Inspection confirmed that the proximal tip of the metallic stent was embedded in the renal pelvic mucosa. Grasp-and-traction attempts failed to mobilize the stent. Therefore, a Ho:YAG laser (frequency 8-10 Hz, energy 0.8-1.0 J/pulse, 365-μm fiber) was used to incise the overlying mucosa; however, removal remained difficult because the embedding was more extensive than expected. An additional incision with 3-mm pediatric laparoscopic scissors freed the remaining adherent tissue, allowing intact removal of the stent with gentle traction (Video 1).

**Video 1:** Operation of nephrostomy-assisted multimodal removal of an embedded ureteral metallic stent.

An antegrade 6-Fr polyurethane DJ ureteral stent and a 20-Fr nephrostomy balloon catheter were then placed. The postoperative course was uneventful, with no fever, sepsis, or flank pain. Preoperative serum creatinine was 0.77 mg/dL (within our institutional reference range of 0.46-0.79 mg/dL) and remained stable with a slight improvement to 0.73 mg/dL postoperatively, with a corresponding increase in estimated glomerular filtration rate (eGFR) from 64.0 to 67.8 mL/min/1.73 m². The nephrostomy balloon catheter was removed on postoperative day four, and the patient was discharged on postoperative day nine. She remains under follow-up with scheduled exchanges of the polyurethane DJ ureteral stent every three months.

## Discussion

Metallic ureteral stents are increasingly used for MUO because they maintain patency with fewer exchanges than polymeric devices; however, difficult retrievals can occur and may be hazardous when repeated blind retrograde traction is attempted [1-4]. As summarized in previous series, metallic ureteral stents for MUO achieve technical or clinical success in approximately 65-88% of cases with higher one-year patency and lower occlusion rates than polymeric DJ ureteral stents [1-3]. This case underscores three practical considerations relevant to routine care: (i) imaging-based assessment of the underlying mechanism before repeat removal; (ii) early transition to a controlled antegrade route when retrograde removal appears unsafe; and (iii) stepwise soft-tissue release, laser incision followed by fine mechanical dissection, before applying definitive traction [5-7].

When a metallic stent cannot be mobilized, distinguishing bulky calcific encrustation from mucosal embedding or intramural migration is critical because each mechanism dictates distinct access and tools [4]. In this patient, non-contrast CT indicated proximal-coil embedding rather than heavy calcification, limiting the benefit of further shock wave lithotripsy and favoring antegrade exposure of the renal pelvis. Beyond standard imaging, a bicentric CT radiomics and machine-learning study demonstrated high diagnostic performance for identifying ureteral stent encrustation, with the combined model achieving accuracies of approximately 78-83% and an area under the receiver operating characteristic curve of 0.82-0.87 in external and internal validation cohorts. CT radiomics involves extracting quantitative texture features from CT images and analyzing them with machine-learning algorithms; however, this currently requires standardized imaging protocols, time-consuming image segmentation, and dedicated software, so it remains an experimental technique available mainly in specialized or research centers rather than a routine option in everyday clinical practice [8]. CT can also identify residual debris after apparent removal, emphasizing the importance of careful image review [9].

Establishing percutaneous antegrade access before removal provides controlled visualization, safe re-orientation of the proximal loop, and immediate hemostasis if needed. Prior reports describe antegrade laser-assisted release and loop-wire maneuvers for retained stents, in line with the multimodal concept applied here [6,7]. The GMSV position further enables simultaneous antegrade and retrograde evaluation and simplifies airway management during prolonged procedures [10].

Energy and instrument selection should favor controlled liberation rather than force. Following this principle, we used low-to-moderate Ho:YAG settings to fenestrate the overlying mucosa, completed fibrotic attachments with fine endoscopic scissors, and applied traction only after circumferential release was confirmed. Prior experience suggests that laser monotherapy may be insufficient in deeply tethered segments and that combining modalities reduces the risk of ureteral or infundibular injury [5-7]. Traction should not increase until a full 360° release is clearly visualized. Historically, deeply embedded ureteral stents have been managed with a range of techniques, including repeated blind retrograde traction, retrograde ureteroscopic Ho:YAG laser cutting, percutaneous nephrolithotomy-type approaches, and, in rare cases, open or laparoscopic explantation [4-7]. Purely retrograde strategies may suffice when embedding is limited, but in extensively tethered proximal coils, they offer restricted visualization and can increase the risk of ureteral or infundibular injury. Conversely, open or laparoscopic removal carries substantial morbidity in patients with advanced malignancy and prior pelvic surgery. Our nephrostomy-assisted multimodal strategy combines the advantages of percutaneous antegrade visualization with graded traction, low-energy Ho:YAG laser release, and fine mechanical dissection, aiming to achieve en bloc explantation while potentially avoiding the need for open surgery in selected cases.

A practical workflow synthesizing available evidence and our experience is as follows: (1) Reassess the patient with non-contrast CT and, when feasible, diagnostic ureteroscopy to differentiate bulky calcific encrustation, mucosal embedding, or intramural migration of the stent [1,4,8,9]. (2) After an initial failed retrograde attempt, avoid further forceful traction and convert early to antegrade access when the proximal coil is involved or visualization is poor [6,7,10]. (3) Perform sequential soft-tissue release, beginning with gentle traction to assess mobility, followed by Ho:YAG laser incision of overlying mucosa and completion of any residual fibrotic attachments with fine endoscopic scissors, and proceed to stent removal only after a complete 360° release is clearly visualized [5-7]. (4) At the end of the procedure, place a temporary DJ ureteral stent and nephrostomy tube to protect the tract, reduce edema-related obstruction, and maintain urinary diversion, particularly in patients with MUO who often require ongoing drainage.

Although this report focuses on retrieval, upstream device choice shapes downstream risk and resource use. Economic modeling suggests that Resonance® metallic stents can reduce repeat procedures and overall costs compared with polymeric DJ ureteral stents in MUO. In a recent UK cost-utility analysis, the metallic stent strategy was associated with roughly four fewer repeat interventions over five years, a 23% reduction in total costs, an estimated cost saving of about £2100 per patient, and a small gain in quality-adjusted life years compared with DJ ureteral stents [11]. Contemporary clinical data also support flexible, anatomy-driven access selection in MUO. In a recent case series of 11 patients undergoing antegrade Resonance® stent placement for chronic malignant ureteral obstruction (16 stents in total), 66.7% of procedures resulted in improved eGFR, stent failure occurred in 25% of stents, and the median indwelling time was 245 days; importantly, antegrade insertion did not significantly shorten dwell time compared with published retrograde cohorts [12]. After successful removal of an embedded metallic stent, ongoing surveillance of renal function and symptoms remains essential, particularly in patients with MUO who require continued urinary diversion. At our institution, follow-up typically includes periodic assessment of serum creatinine and estimated glomerular filtration rate, review of flank pain, fever, and lower urinary tract symptoms, and ultrasonography or CT at the time of subsequent stent exchanges or at three- to six-month intervals, tailored to oncologic prognosis. Clinicians should remain alert for delayed complications such as recurrent obstruction, urinary tract infection or sepsis, hematuria, and iatrogenic ureteral stricture, which may warrant earlier imaging or re-intervention. Combined with the present workflow, these findings support institutional pathways that standardize pre-removal imaging, define early indicators for antegrade conversion, and ensure the availability of laser fibers, fine endoscopic scissors, loop-wire retrieval tools, and GMSV positioning.

This single case involved minimal calcific burden; more extensive encrustation may require staged lithotripsy and multi-site debulking [4,5,8]. As this is a single case report, no formal statistical analysis was performed, and any conclusions regarding efficacy should therefore be interpreted with appropriate caution. Even so, the overarching principle of secure access → controlled release → atraumatic removal appears broadly applicable across mechanisms and devices. Multicenter registries should prospectively capture patient and disease characteristics (primary malignancy, etiology of MUO, prior stent type and dwell time, and the degree and mechanism of embedding or encrustation), procedural variables (access route, imaging modality, energy settings, and adjunctive instruments), and short- and longer-term outcomes (technical and clinical success, need for open conversion, changes in renal function, perioperative and delayed complications, recurrent obstruction, and subsequent stent patency) to refine and validate this algorithm in MUO.

## Conclusions

When metallic ureteral stents cannot be safely removed via standard retrograde techniques, a nephrostomy-assisted multimodal approach combining antegrade access, controlled traction, Ho:YAG laser incision, and endoscopic scissor dissection may allow for effective and safe en bloc removal while preserving ureteral integrity. Although this report describes a single case and cannot establish comparative efficacy, it demonstrates the potential effectiveness and safety of this strategy and may provide a practical option in similarly challenging embedded stent scenarios. Larger series and multicenter registries will be required to validate this approach, define its indications and contraindications, and refine the proposed workflow.
